# Supplementary material for: Human TRPC5 structures reveal interaction of a xanthine-based TRPC1/4/5 inhibitor with a conserved lipid binding site
Source: Commun Biol. 2020 Nov 23;3:704. doi: 10.1038/s42003-020-01437-8 (PMC7683545; doi:10.1038/s42003-020-01437-8)
Supplement: Supplementary file 1 — Supplementary Information [file 42003_2020_1437_MOESM1_ESM.pdf]

# Supplementary Information for:

## Human TRPC5 structures reveal interaction of a xanthine-based TRPC1/4/5 inhibitor with a conserved lipid binding site.

David J. Wright,[1,3] Katie J. Simmons,[1,3] Rachel M. Johnson,[2,3] David J. Beech,[1] Stephen P. Muench,[2,3,*] and Robin S. Bon[1,3,*]

*[1] Leeds Institute of Cardiovascular and Metabolic Medicine, LIGHT Laboratories, University of Leeds, Leeds LS2 9JT, UK.*

*[2] School of Biomedical Sciences, University of Leeds, Woodhouse Lane, Leeds LS2 9JT, UK.*

*[3] Astbury Centre for Structural Molecular Biology, University of Leeds, Woodhouse Lane, Leeds LS2 9JT, UK.*

** Correspondence should be addressed to R.S.B. (r.bon@leeds.ac.uk) or S.P.M. (s.p.muench@leeds.ac.uk)*

## Supplementary Figures

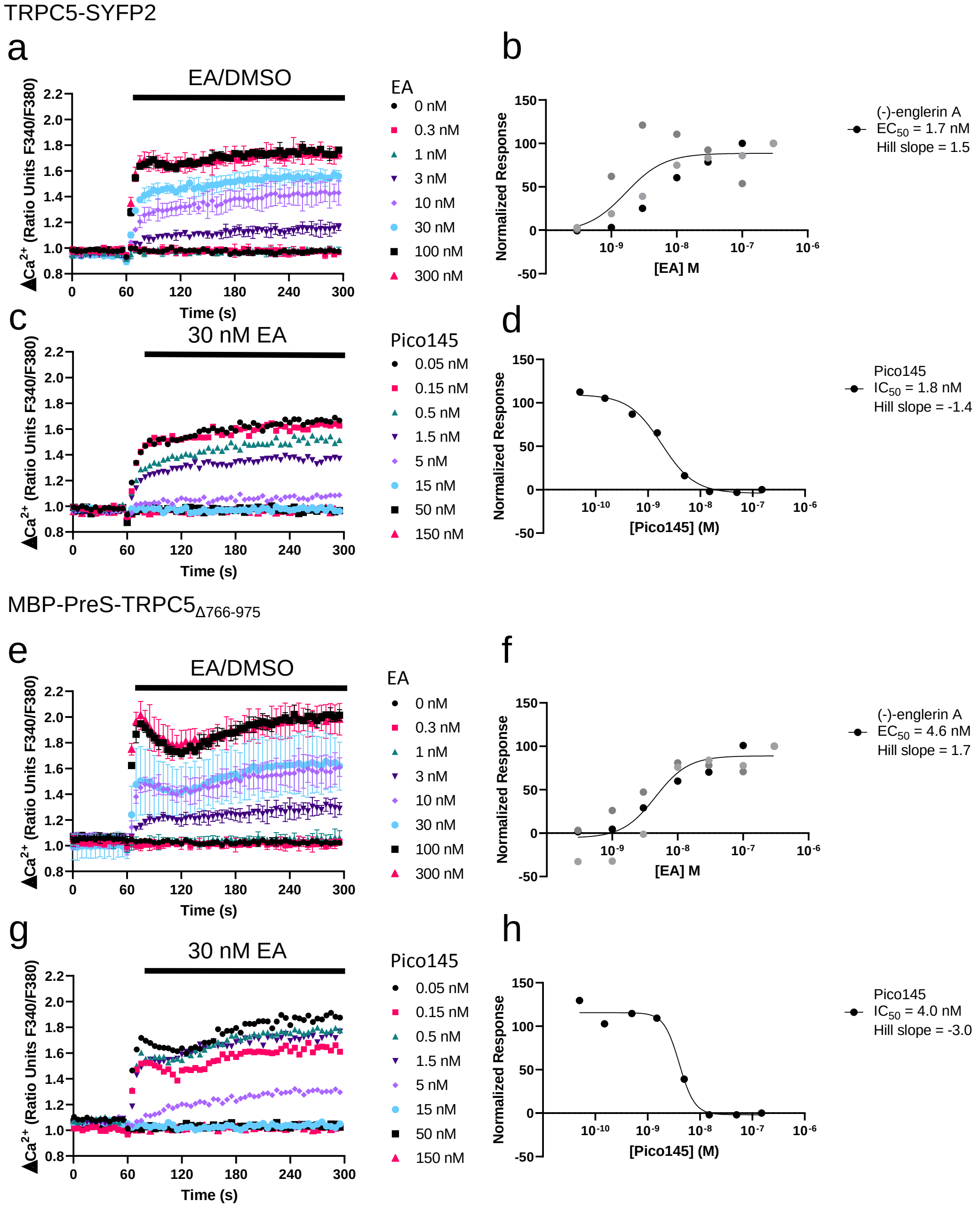


**Supplementary Figure 1:** Response of C-terminally truncated, N-terminally labelled hTRPC5 to EA and Pico145 is comparable with that of full-length hTRPC5-SYFP2. a,e) Representative traces from single 96-well plates (N = 2; error bars show standard deviations over technical repeats) showing an increase in $[Ca^{2+}]_i$ in response to 0.32 nM to 5 µM EA of HEK 293 cells transiently expressing TRPC5-SYFP (a) or MBP-PreS-TRPC5$_{\Delta 766\text{-}975}$ (e). b,f) Concentration-response data for experiments (a) and (e) (mean normalised response ± SEM; n/N = 3/8). c,g) Traces from single 96-well plates (N = 1) showing that the increase in $[Ca^{2+}]_i$ in response to 30 nM EA is concentration-dependently inhibited by pre-incubation with 0.05 nM to 150 nM Pico145 in HEK 293 cells transiently expressing TRPC5-SYFP (c) or MBP-PreS-TRPC5$_{\Delta 766\text{-}975}$ (g). f,h) Concentration-response data for experiments in (c) and (g). Responses were calculated at 240-300 s compared to $[Ca^{2+}]_i$ at baseline (0–60 s). Averages of technical repeats (where relevant) of each individual experiment were normalised to the maximum and minimum response for each set of experiments, combined, and fit with GraphPad Prism 8 (variable slope, four-parameters fit).

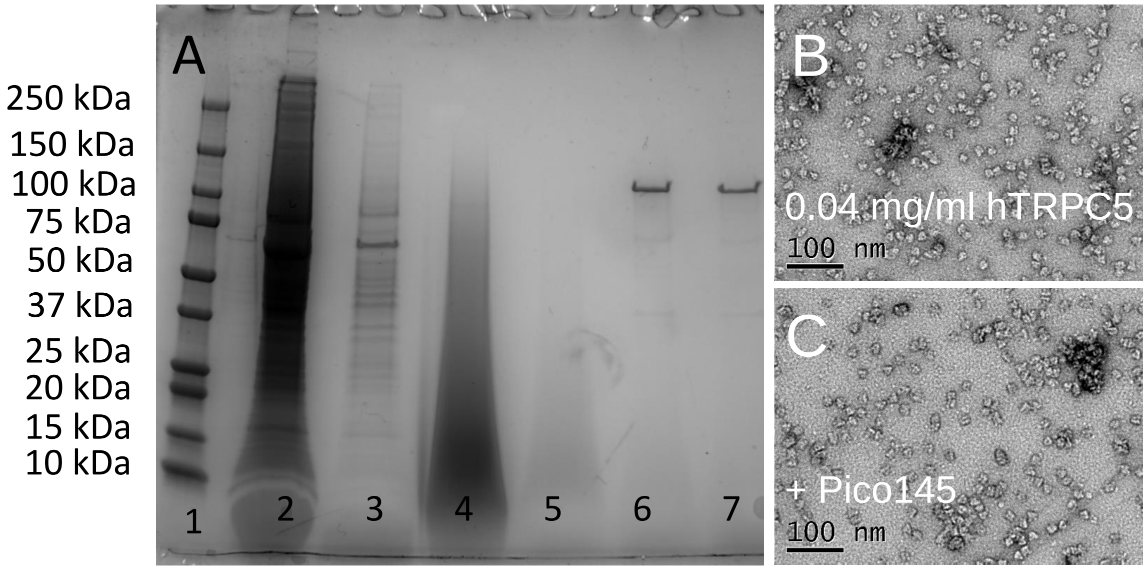


**Supplementary Figure 2:** Purification of MBP-PreS-TRPC5$_{\Delta766\text{-}975}$ and negative stain electron microscopy. a) Purification of hTRPC5 using amylose resin and exchange into PMAL-C8 amphipol. Lanes: **1.** Ladder; **2.** Flow-through A; **3.** Wash A; **4.** Flow-through after Amphipol exchange; **5.** Wash B; **6.** Elution; **7.** Supernatant of ultracentrifugation. b) Negative stain electron microscopy of purified hTRPC5 at 49,000 x magnification. c) Negative stain electron microscopy of purified hTRPC5 (0.04 mg·ml$^{-1}$) at 49,000 x magnification, plus 4 µM Pico145.

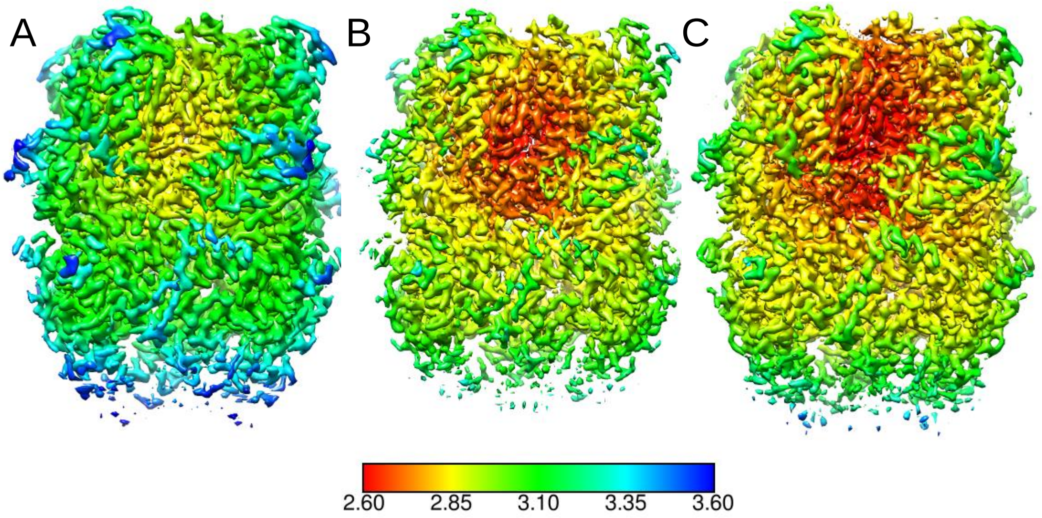


**Supplementary Figure 3:** Local resolution of TRPC5 structures. a) Local resolution of the hTRPC5:Pico145 structure (contour level: 0.0569). b) Local resolution of the partially Pico145-occupied hTRPC5 structure (contour level: 0.0979). c) Local resolution of the hTRPC5 structure in the presence of 20 μM $ZnCl_2$ (contour level: 0.0653).

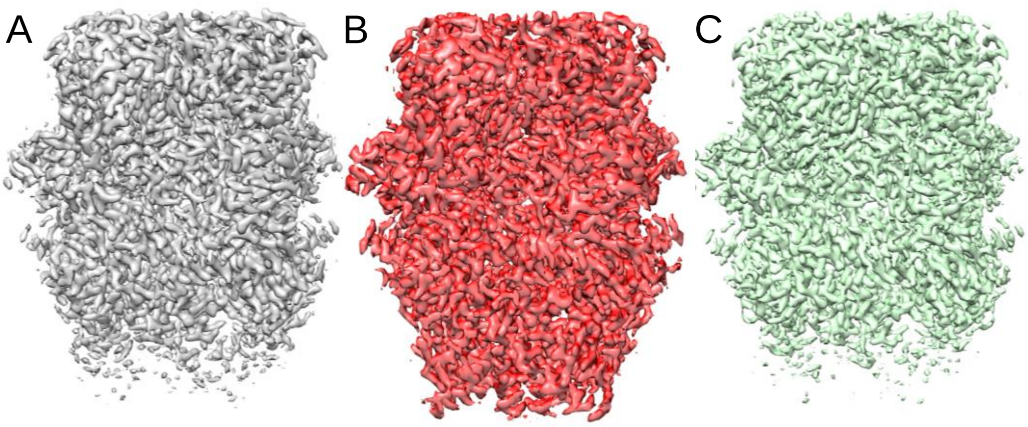


**Supplementary Figure 4:** Comparison of overall TRPC5 structures. a) Electron density of hTRPC5 in the presence of 100 µM Pico145 (contour level: 0.0373). b) Electron density of mTRPC5 *apo* (PDB 6aei; EMDB 9615; contour level: 0.0510). c) Electron density of hTRPC5 structure in the presence of 20 µM $ZnCl_2$ (contour level: 0.0471).

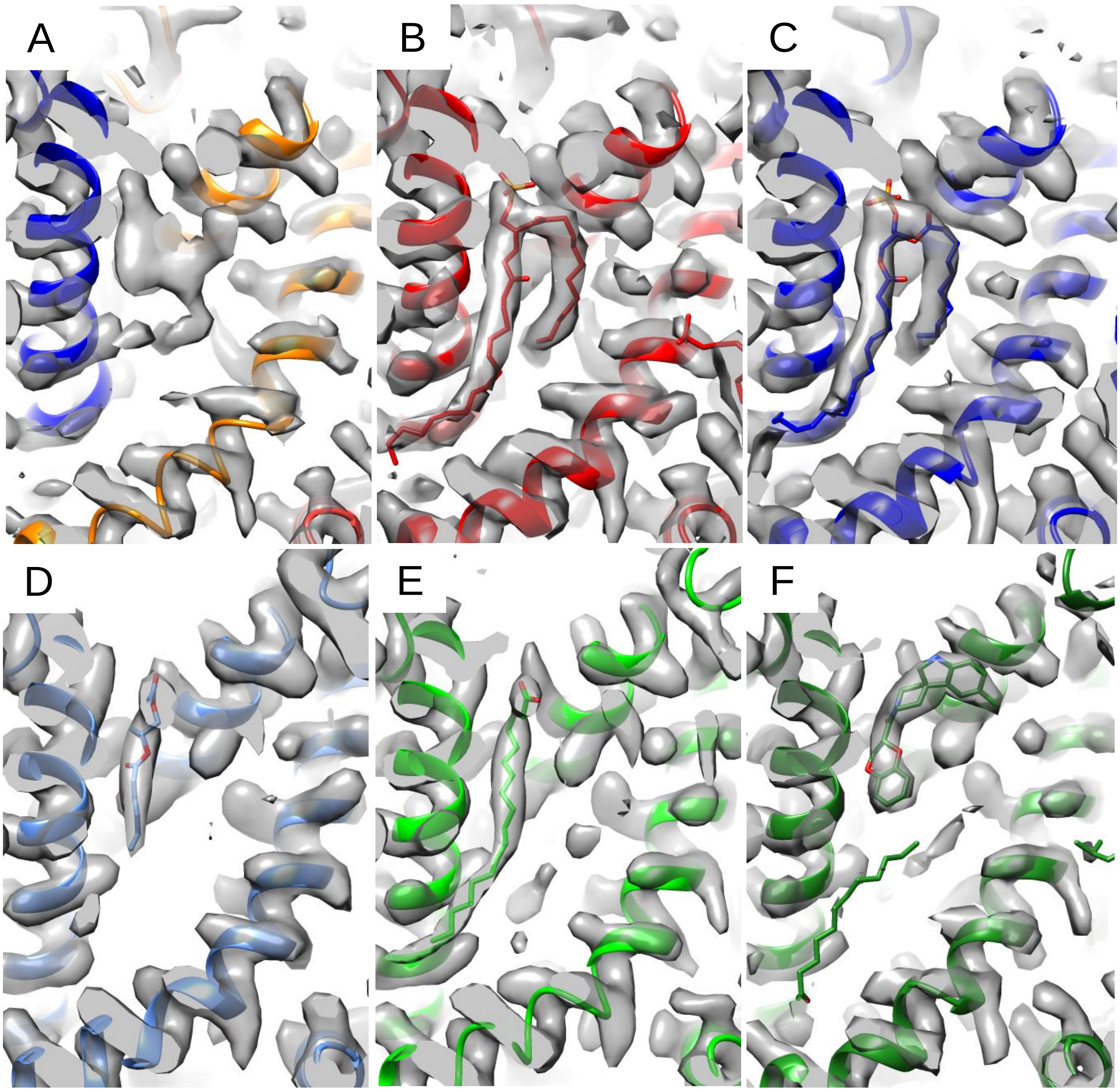


**Supplementary Figure 5:** Pico145 binds to a conserved TRPC4/5 lipid binding site. a) Density observed in Pico145-bound hTRPC5 structure (100 µM Pico145; contour level: 0.0621). b) Equivalent density in the published mTRPC5 *apo* structure (PDB 6aei; EMDB 9615) that was modelled as a phospholipid (contour level: 0.0510). c) The same site from the published mTRPC4 structure (PDB 5z96; EMDB 6901; contour level: 0.0331). d) Lipid bound to equivalent residues in the hTRPC3 structure (PDB 6cud; EMDB 7620; contour level: 0.0414). e) The equivalent site from the structure of hTRPC6 in complex with the TRPC6 antagonist AM-1473 (PDB 6uz8; EMDB 20953) revealing a bound lipid (contour level: 7.33). f) The same site from the published structure of hTRPC6 in complex with the TRPC6 agonist AM-0883 (PDB 6uza; EMDB 20954), showing the displacement of lipid by the small molecule AM-0883 (contour level: 0.0421).

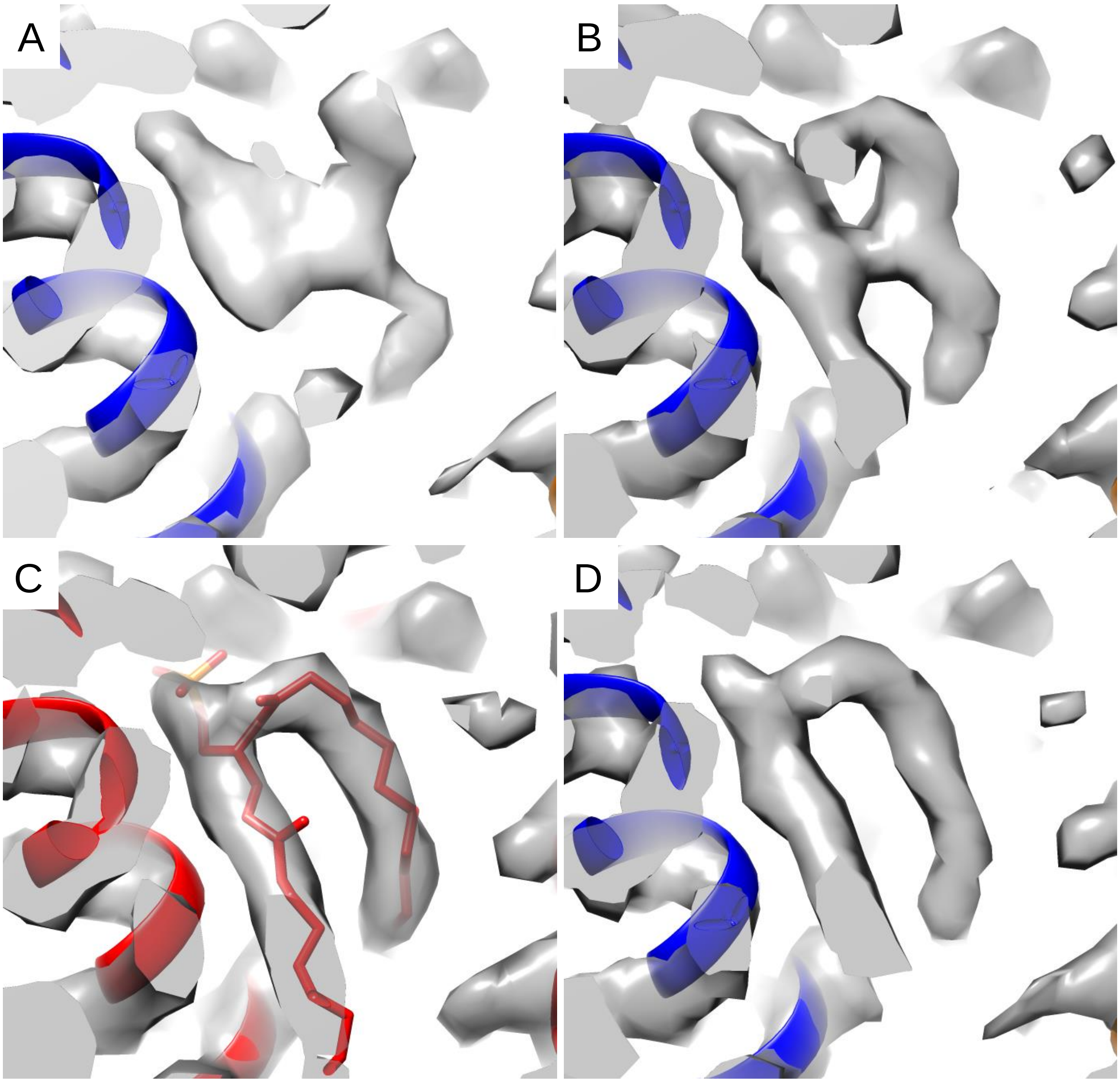


**Supplementary Figure 6:** Comparison of the Pico145/lipid binding site of TRPC5 in three structures. a) Lipid/xanthine binding site of hTRPC5 in the presence of 100 µM Pico145 (contour level: 0.0707). b) Lipid/xanthine binding site of hTRPC5 in the presence of 50 µM Pico145 (contour level: 0.0458. c) Lipid/xanthine binding site of mTRPC5 *apo* (PDB 6aei; EMDB 9615; contour level: 0.0510). d) Lipid/xanthine binding site of hTRPC5 in the presence of 20 µM $ZnCl_2$ (contour level: 0.0471).

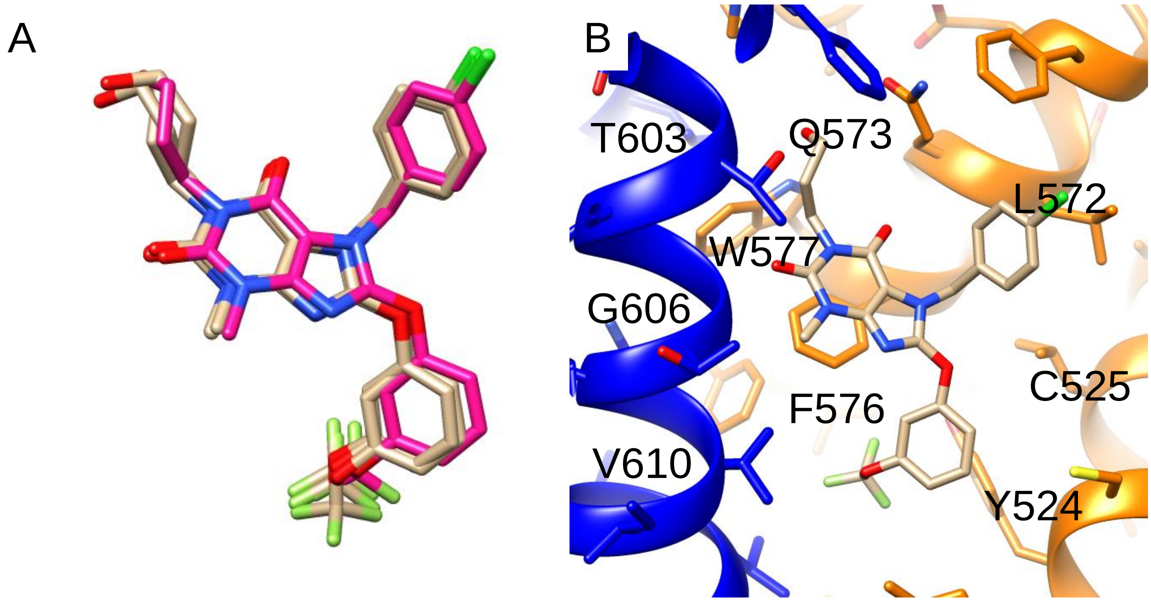


**Supplementary Figure 7:** Docking of Pico145 into hTRPC5 shows key interactions. a) The top three docked poses of Pico145 in TRPC5 (peach) overlaid with the refined structure (magenta) in the absence of the protein structure. There is good agreement between the structures and the position of the xanthine core is highly conserved. The different positions of the flexible 3-hydroxypropyl substituent at N-1 suggest it is flexible within the TRPC5 binding pocket. b) The best scoring docking pose is shown relative to the PDB file used for docking (as in Figure 3).

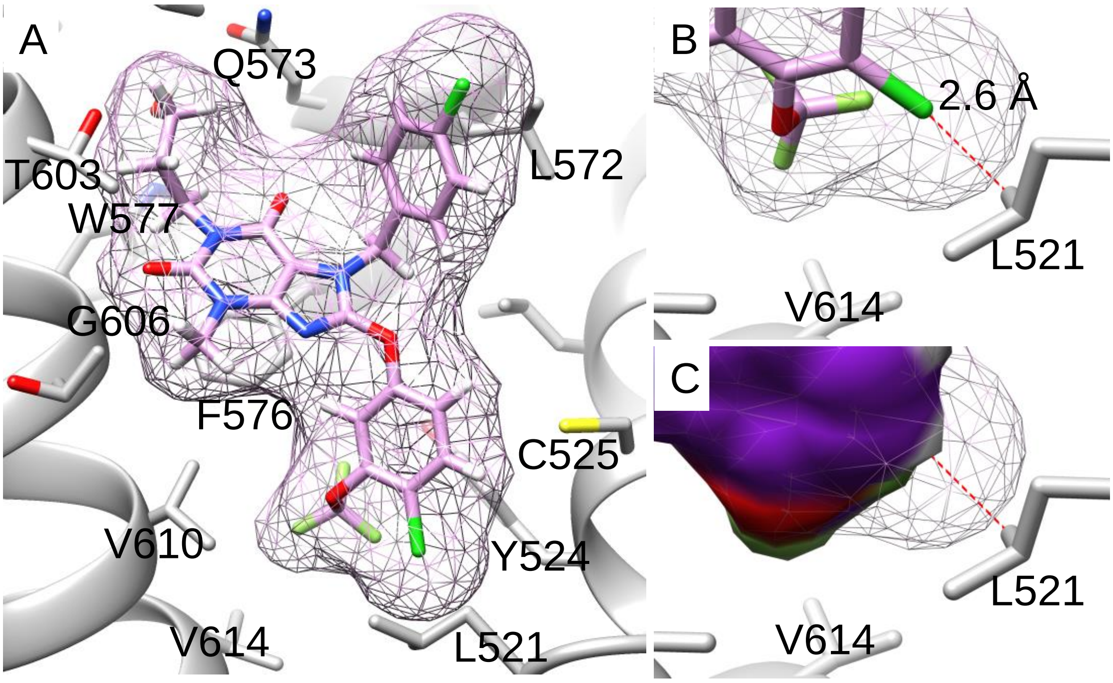


**Supplementary Figure 8:** AM237 in the same pose as Pico145 in TRPC5 would clash with L521. AM237 was modelled into the top scoring docking pose of Pico145 in TRPC5. a) The full AM237 structure is shown in mesh format. b) A zoomed in view of the clash between chlorine atom and L521. c) The same view as in (b), but with the surface of docked Pico145 structure shown as solid colour, showing the removal of the clash with L521.

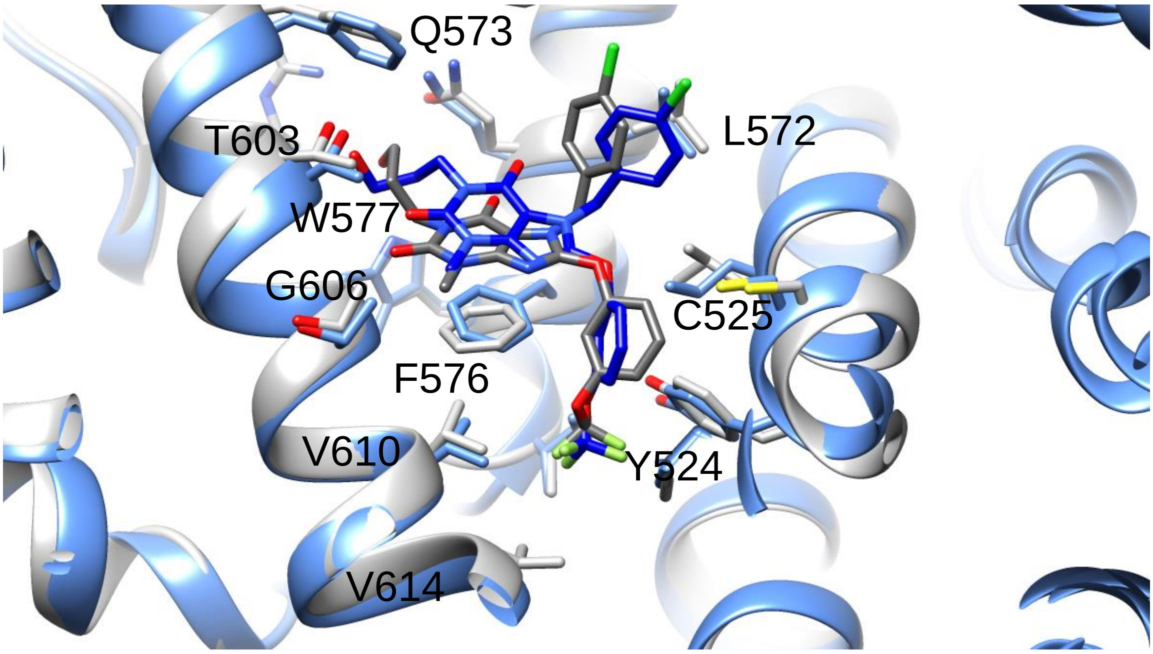


**Supplementary Figure 9:** The docked poses of Pico145 in TRPC5 and TRPC4 are similar. Pico145 was docked into TRPC5 (grey: our TRPC5:Pico145 structure minus refined Pico145) and TRPC4 (PDB 5z96; blue). All interacting residues are conserved (**Supplementary Table 1**) except V579 and the docking poses overlay well. Only the top scoring docking pose (of three) is shown for each TRPC structure.

Wild-type HEK 293 cells

A

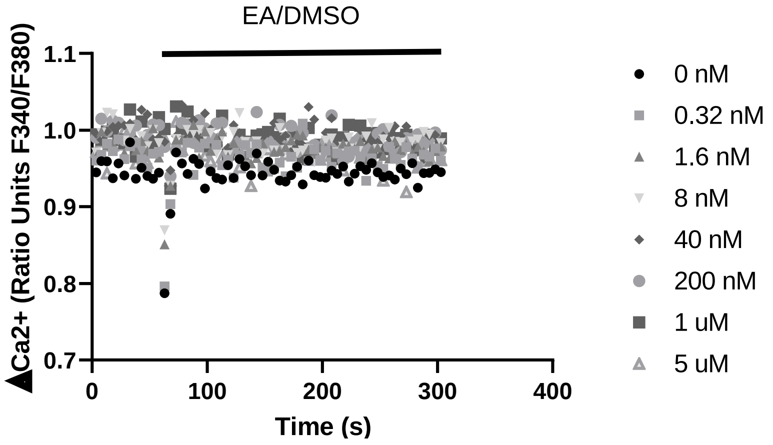


B

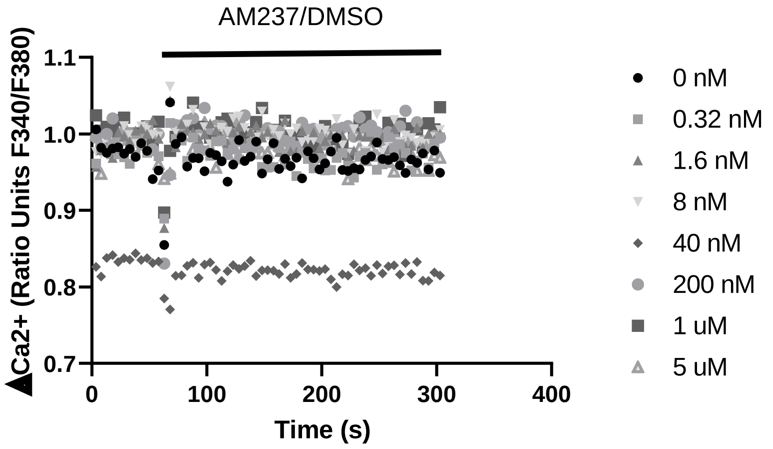


**Supplementary Figure 10:** The TRPC5 activators EA and AM237 do not cause non-specific calcium signals in wild-type HEK 293 cells. a) $[Ca^{2+}]_i$ measurements from a single 96-well plate (N = 1) showing that 0.3-5000 nM EA has no effect on $[Ca^{2+}]_i$ in wild-type HEK 293 cells. b) $[Ca^{2+}]_i$ measurements from a single 96-well plate (N = 1) showing that 0.3-5000 nM AM237 has no effect on $[Ca^{2+}]_i$ in wild-type HEK 293 cells.

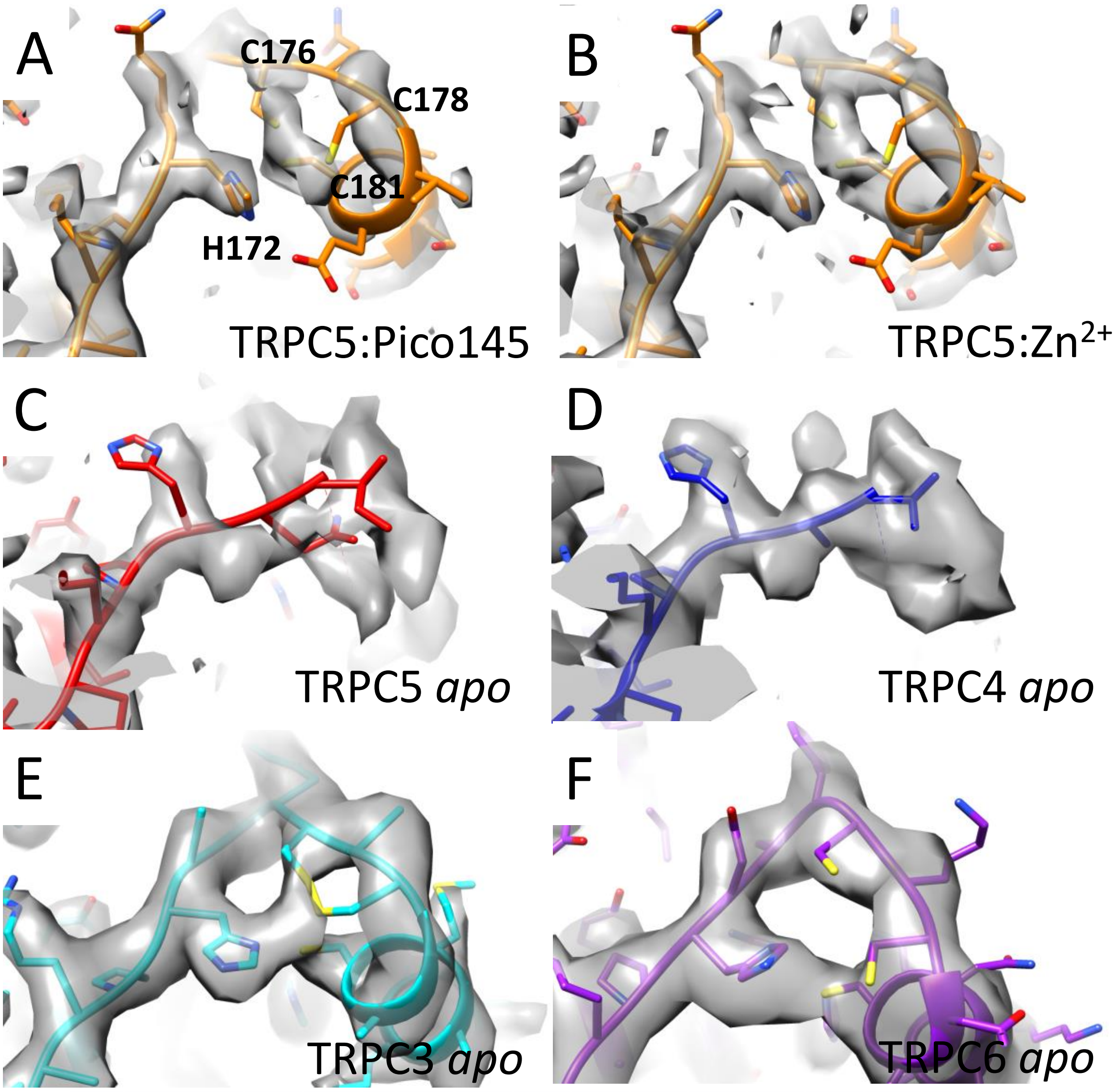


| G | Starting Residue | | | H172 | | | | C176 | | C178 | | | C181 | | Final residue |
|---|---|---|---|---|---|---|---|---|---|---|---|---|---|---|---|
| TRPC5 | 170 | R | P | H | Q | I | R | C | N | C | V | E | C | V | 182 |
| TRPC4 | 170 | R | P | H | E | V | R | C | N | C | V | E | C | V | 182 |
| TRPC1 | 187 | K | P | H | A | V | G | C | E | C | T | L | C | S | 199 |
| TRPC3 | 261 | R | P | H | D | Y | F | C | K | C | G | D | C | M | 273 |
| TRPC7 | 192 | R | P | H | D | Y | F | C | K | C | N | E | C | T | 204 |
| TRPC6 | 247 | R | P | H | D | Y | F | C | K | C | N | D | C | N | 259 |

**Supplementary Figure 11:** Alignment of the putative zinc binding sites of TRPC channels. a-f) The putative zinc binding site of hTRPC5 in the presence of 2 µM Pico145 (a) (contour level: 0.03) and hTRPC5 in the presence of 20 µM $ZnCl_2$ (b) (contour level: 0.03), mTRPC5 (PDB 6aei; EMDB 9615; (c); contour level: 0.05), mTRPC4 (PDB 5z96; EMDB 6901; (d); contour level: 0.03), hTRPC3 (PDB 6cud; EMDB 7620; (e); contour level: 0.04) and hTRPC6 (PDB 5yx9; EMDB 6856; (f); contour level: 0.05) is shown with PDB files fit into experimental densities. g) Sequence alignment of hTRPC5 with the other human TRPC proteins, showing conservation of His and Cys residues in the putative zinc binding sites.

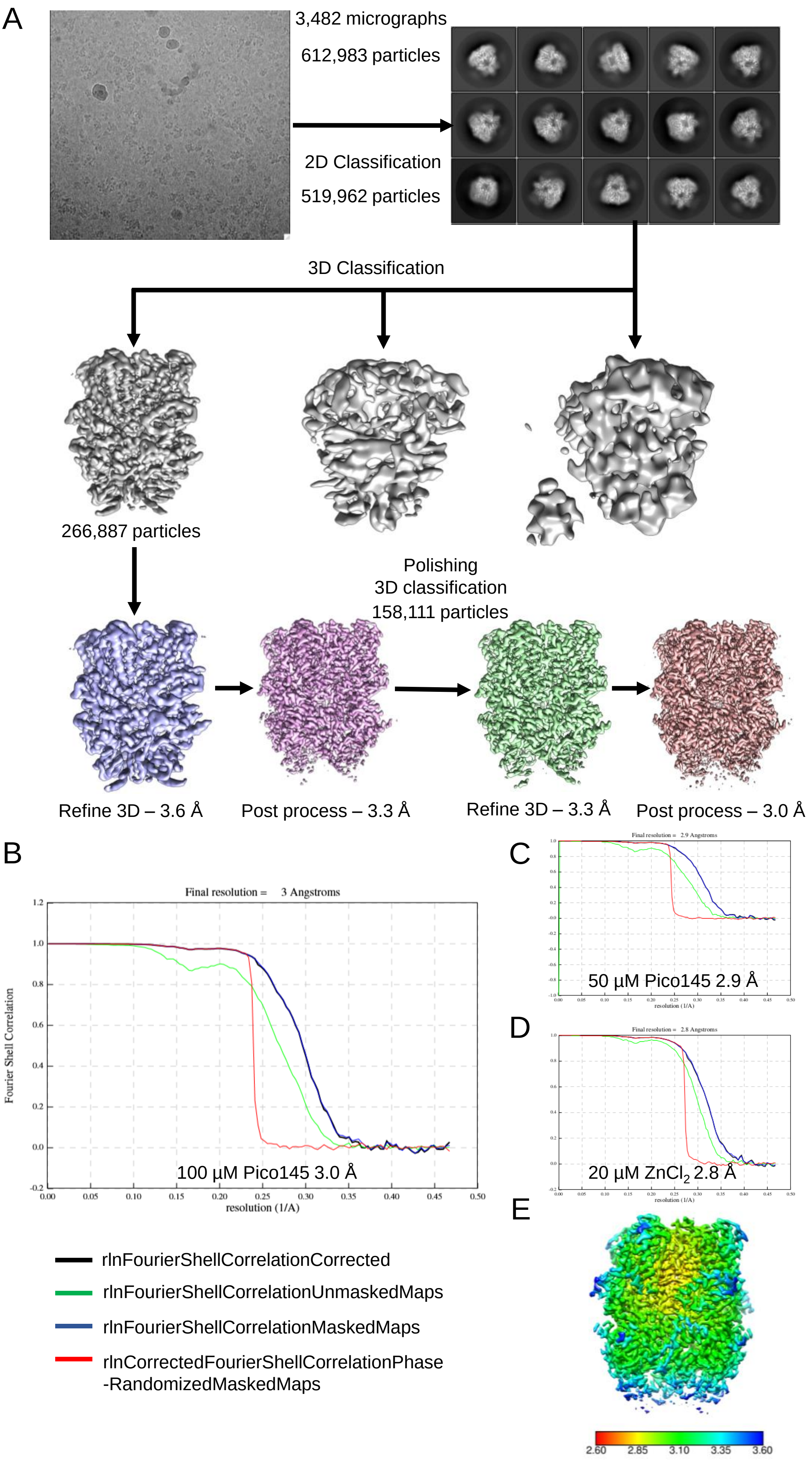
A
3,482 micrographs
612,983 particles
2D Classification
519,962 particles
3D Classification
266,887 particles
Polishing
3D classification
158,111 particles
Refine 3D – 3.6 Å
Post process – 3.3 Å
Refine 3D – 3.3 Å
Post process – 3.0 Å
B
Final resolution = 3 Angstroms
Fourier Shell Correlation
resolution (1/A)
100 µM Pico145 3.0 Å
rlnFourierShellCorrelationCorrected
rlnFourierShellCorrelationUnmaskedMaps
rlnFourierShellCorrelationMaskedMaps
rlnCorrectedFourierShellCorrelationPhase
-RandomizedMaskedMaps
C
Final resolution = 2.9 Angstroms
50 µM Pico145 2.9 Å
D
Final resolution = 2.8 Angstroms
20 µM $ZnCl_2$ 2.8 Å
E
2.60
2.85
3.10
3.35
3.60

**Supplementary Figure 12:** TRPC5 Cryo-EM data processing. a) Representative micrograph of grids prepared from 2.0 mg·ml$^{-1}$ MBP-PreS-hTRPC5$_{\Delta 766\text{-}975}$ with 100 µM Pico145, and graphical representation of 2D and 3D classification, refinement and post-processing as described in the Methods section. b) Fourier shell correlation (FSC) curves for the 3D reconstruction from grids prepared from 2.0 mg·ml$^{-1}$ of MBP-PreS-hTRPC5$_{\Delta 766\text{-}975}$ with 100 µM Pico145 (3.0 Å resolution). c) FSC curves for the 3D reconstruction from grids prepared from 1.0 mg·ml$^{-1}$ MBP-PreS-hTRPC5$_{\Delta 766\text{-}975}$ with 50 µM Pico145 (2.9 Å resolution). d) FSC curves for the 3D reconstruction from grids prepared from 1.0 mg·ml$^{-1}$ MBP-PreS-hTRPC5$_{\Delta 766\text{-}975}$ with 20 µM ZnCl2 (2.8 Å resolution). e) Local resolution estimation from Relion.

## Supplementary Tables

**Supplementary Table 1:** Sequence alignment of xanthine binding site within the TRPC family. Residues 520-616 of TRPC5 were aligned within the human TRPC proteins. Overall identity to TRPC5 is shown and residues equivalent to those close to Pico145 in the TRPC5 structure are shaded in orange (monomer 1; **Figure 3A**) and blue (monomer 2). The numbers above residues above are those in TRPC5. TRPC1, TRPC4 and TRPC5 are coloured in red because channels containing these monomers can be modulated by xanthines such as Pico145 and AM237.

| Channel (identity to TRPC5) | | L521 | | | Y524 | C525 | | | L528 | | | | | | | | | | | | | | | | |
|---|---|---|---|---|---|---|---|---|---|---|---|---|---|---|---|---|---|---|---|---|---|---|---|---|---|
| TRPC5 | F | L | F | I | Y | C | L | V | L | L | A | F | A | N | G | L | N | Q | L | Y | F | Y | Y | E | T |
| TRPC4 (70%) | F | L | F | I | Y | C | L | V | L | L | A | F | A | N | G | L | N | Q | L | Y | F | Y | Y | E | E |
| TRPC1 (47%) | F | L | G | M | F | L | L | V | L | F | S | F | T | I | G | L | T | Q | L | Y | D | K | G | Y | T |
| TRPC3 (39%) | F | M | V | L | F | I | M | V | F | F | A | F | M | I | G | M | F | I | L | Y | S | Y | Y | - | - |
| TRPC7 (40%) | F | M | V | I | F | I | M | V | F | V | A | F | M | I | G | M | F | N | L | Y | S | Y | Y | - | - |
| TRPC6 (37%) | F | M | V | I | F | I | M | V | F | V | A | F | M | I | G | M | F | N | L | Y | S | Y | Y | - | - |

| Channel (identity to TRPC5) | | | | | | | | | | K554 | | | R557 | | | | | | | | | | | | |
|---|---|---|---|---|---|---|---|---|---|---|---|---|---|---|---|---|---|---|---|---|---|---|---|---|---|
| TRPC5 | R | A | I | D | E | P | N | N | C | K | G | I | R | C | E | K | Q | - | N | N | A | F | S | T | L |
| TRPC4 (70%) | T | - | - | - | K | G | L | T | C | K | G | I | R | C | E | K | Q | - | N | N | A | F | S | T | L |
| TRPC1 (47%) | S | K | - | - | E | Q | K | D | C | V | G | I | F | C | E | Q | Q | S | N | D | T | F | H | S | F |
| TRPC3 (39%) | - | - | - | - | - | - | - | - | - | - | - | - | L | G | A | K | V | - | N | A | A | F | T | T | V |
| TRPC7 (40%) | - | - | - | - | - | - | - | - | - | - | - | - | R | G | A | K | Y | - | N | P | A | F | T | T | V |
| TRPC6 (37%) | - | - | - | - | - | - | - | - | - | - | - | - | I | G | A | K | Q | - | N | E | A | F | T | T | V |

| Channel (identity to TRPC5) | | | | L572 | Q573 | | | F576 | W577 | | V579 | | | | | | | | | | | | | | |
|---|---|---|---|---|---|---|---|---|---|---|---|---|---|---|---|---|---|---|---|---|---|---|---|---|---|
| TRPC5 | F | E | T | L | Q | S | L | F | W | S | V | F | G | L | L | N | L | Y | V | T | N | V | K | A | R |
| TRPC4 (70%) | F | E | T | L | Q | S | L | F | W | S | I | F | G | L | I | N | L | Y | V | T | N | V | K | A | Q |
| TRPC1 (47%) | I | G | T | C | F | A | L | F | W | Y | I | F | S | L | A | H | V | A | I | F | V | T | R | F | S |
| TRPC3 (39%) | E | E | S | F | K | T | L | F | W | S | I | F | G | L | S | E | V | T | S | V | V | L | K | Y | D |
| TRPC7 (40%) | E | E | S | F | K | T | L | F | W | S | I | F | G | L | S | E | V | I | S | V | V | L | K | Y | D |
| TRPC6 (37%) | E | E | S | F | K | T | L | F | W | A | I | F | G | L | S | E | V | K | S | V | V | I | N | Y | N |

| Channel (identity to TRPC5) | | | | | | | | F599 | | | | T603 | | | G606 | T607 | | | V610 | | | | V614 | | |
|---|---|---|---|---|---|---|---|---|---|---|---|---|---|---|---|---|---|---|---|---|---|---|---|---|---|
| TRPC5 | H | - | - | E | F | T | E | F | V | G | A | T | M | F | G | T | Y | N | V | I | S | L | V | V | L |
| TRPC4 (70%) | H | - | - | E | F | T | E | F | V | G | A | T | M | F | G | T | Y | N | V | I | S | L | V | V | L |
| TRPC1 (47%) | Y | G | E | E | L | Q | S | F | V | G | A | V | I | V | G | T | Y | N | V | V | V | V | I | V | L |
| TRPC3 (39%) | H | - | - | K | F | I | E | N | I | G | Y | V | L | Y | G | I | Y | N | V | T | M | V | V | V | L |
| TRPC7 (40%) | H | - | - | K | F | I | E | N | I | G | Y | V | L | Y | G | V | Y | N | V | T | M | V | V | V | L |
| TRPC6 (37%) | H | - | - | K | F | I | E | N | I | G | Y | V | L | Y | G | V | Y | N | V | T | M | V | I | V | L |
