# Supplementary material for: Human TRPC5 structures reveal interaction of a xanthine-based TRPC1/4/5 inhibitor with a conserved lipid binding site
Source: Commun Biol. 2020 Nov 23;3:704. doi: 10.1038/s42003-020-01437-8 (PMC7683545; doi:10.1038/s42003-020-01437-8)
Supplement: Supplementary file 3 — Description of Additional Supplementary Files [file 42003_2020_1437_MOESM3_ESM.docx]

Description of Additional Supplementary Files

Supplementary Data 1: Raw data for intracellular calcium recordings (**Figure 4**)
