# Supplementary material for: Human TRPC5 structures reveal interaction of a xanthine-based TRPC1/4/5 inhibitor with a conserved lipid binding site
Source: Commun Biol. 2020 Nov 23;3:704. doi: 10.1038/s42003-020-01437-8 (PMC7683545; doi:10.1038/s42003-020-01437-8)
Supplement: Supplementary file 5 — Reporting Summary [file 42003_2020_1437_MOESM5_ESM.pdf]

nature research

Corresponding author(s): Robin S Bon and Stephen P Muench

Last updated by author(s): Oct 19, 2020

Reporting Summary

Nature Research wishes to improve the reproducibility of the work that we publish. This form provides structure for consistency and transparency in reporting. For further information on Nature Research policies, see our Editorial Policies and the Editorial Policy Checklist.

Statistics

For all statistical analyses, confirm that the following items are present in the figure legend, table legend, main text, or Methods section.

n/a	Confirmed	
☐	☒	The exact sample size (*n*) for each experimental group/condition, given as a discrete number and unit of measurement
☐	☒	A statement on whether measurements were taken from distinct samples or whether the same sample was measured repeatedly
☒	☐	The statistical test(s) used AND whether they are one- or two-sided<br>*Only common tests should be described solely by name; describe more complex techniques in the Methods section.*
☒	☐	A description of all covariates tested
☒	☐	A description of any assumptions or corrections, such as tests of normality and adjustment for multiple comparisons
☐	☒	A full description of the statistical parameters including central tendency (e.g. means) or other basic estimates (e.g. regression coefficient) AND variation (e.g. standard deviation) or associated estimates of uncertainty (e.g. confidence intervals)
☒	☐	For null hypothesis testing, the test statistic (e.g. *F*, *t*, *r*) with confidence intervals, effect sizes, degrees of freedom and *P* value noted<br>*Give P values as exact values whenever suitable.*
☒	☐	For Bayesian analysis, information on the choice of priors and Markov chain Monte Carlo settings
☒	☐	For hierarchical and complex designs, identification of the appropriate level for tests and full reporting of outcomes
☒	☐	Estimates of effect sizes (e.g. Cohen's *d*, Pearson's *r*), indicating how they were calculated

Our web collection on statistics for biologists contains articles on many of the points above.

Software and code

Policy information about availability of computer code

Data collection	Cryo-EM data were collected using the standard EPU data collection software installed by FEI (EPU 2.2 and Digital Micrograph 3.23). Intracellular calcium measurements used the SoftMax Pro 7 software.
Data analysis	Data were analysed using open access software, as described in the Materials and Methods section. Data were CTF corrected in MotionCorr2, particles were picked in crYOLO 1.3.5 and data processed in RELION 3.0.7. Model building was done using WinCoot and refinement in PHENIX (1.16). Model validation, as reported in the paper was conducted using PHENIX and Molprobity. UCSF Chimera (1.13.1) was used for visualisation and figure preparation. Analysis of data from intracellular calcium measurements was performed using GraphPad Prism 8. For docking experiments, Glide (Schrödinger Release 2019-4), Maestro and OMEGA (2.5.1.4) were used.

For manuscripts utilizing custom algorithms or software that are central to the research but not yet described in published literature, software must be made available to editors and reviewers. We strongly encourage code deposition in a community repository (e.g. GitHub). See the Nature Research guidelines for submitting code & software for further information.

Data

Policy information about availability of data

All manuscripts must include a data availability statement. This statement should provide the following information, where applicable:

- Accession codes, unique identifiers, or web links for publicly available datasets
- A list of figures that have associated raw data
- A description of any restrictions on data availability

Structural data are available via PDB 6YSN, EMDB 10903, EMDB 10909 and EMDB 10910. Example raw data for intracellular calcium recordings are displayed in Figure 4 and Supplementary Figure 1. Other data and materials are available from the corresponding authors upon reasonable request.

Field-specific reporting

Please select the one below that is the best fit for your research. If you are not sure, read the appropriate sections before making your selection.

☒ Life sciences ☐ Behavioural & social sciences ☐ Ecological, evolutionary & environmental sciences

For a reference copy of the document with all sections, see nature.com/documents/nr-reporting-summary-flat.pdf

Life sciences study design

All studies must disclose on these points even when the disclosure is negative.

Sample size	The aim of the study was to decipher the mode of action of a pharmacological tool through structural studies. Intracellular calcium measurements were used to: 1) show that expression of the TRPC5 construct used in our structural work resulted in functional TRPC5 channels that retained sensitivity to (-)-englerin A and Pico145; 2) show that expression of the TRPC5 point mutants generated in this work led to functional TRPC5 channels that retained sensitivity to (-)-englerin A; and 3) to support the hypothesis that the mutated residues are important for the modulation of TRPC5 channels by xanthines such as AM237. Sample size calculations were not considered applicable to these studies. The number of repeats used was deemed sufficient to support the conclusions drawn.
Data exclusions	No data were excluded.
Replication	The number of independent repeats (n) and number of total repeats (N) used for intracellular calcium measurements are stated in the legends of Figure 4 and Supplementary Figure 1. There were no outliers and no data were excluded. The number of repeats used was deemed sufficient to support the conclusions drawn.
Randomization	N/A; no clinical samples or animal samples were used
Blinding	N/A; all samples for intracellular calcium measurements were prepared together and read automatically in the SoftMax Pro Software.

Reporting for specific materials, systems and methods

We require information from authors about some types of materials, experimental systems and methods used in many studies. Here, indicate whether each material, system or method listed is relevant to your study. If you are not sure if a list item applies to your research, read the appropriate section before selecting a response.

Materials & experimental systems

n/a	Involved in the study	
☒	☐	Antibodies
☐	☒	Eukaryotic cell lines
☒	☐	Palaeontology and archaeology
☒	☐	Animals and other organisms
☒	☐	Human research participants
☒	☐	Clinical data
☒	☐	Dual use research of concern

Methods

n/a	Involved in the study	
☒	☐	ChIP-seq
☒	☐	Flow cytometry
☒	☐	MRI-based neuroimaging

Eukaryotic cell lines

Policy information about cell lines

Cell line source(s)	HEK 293 (ATCC) and Freestyle 293F (ThermoFisher Scientific).
Authentication	None of the cell lines used were authenticated.
Mycoplasma contamination	The cell lines were not tested for mycoplasma contamination.
Commonly misidentified lines (See ICLAC register)	HEK 293 cells are used routinely by the investigators (and others in the field) for intracellular calcium measurements upon transient expression of TRPC channels (e.g., Minard et al., Br. J. Pharmacol. 2019; Rubaiy et al., J. Biol. Chem. 2017; Naylor et al., Br. J. Pharmacol. 2016). Before transfection, these cells do not give a calcium response upon treatment with (-)-englerin A, suggesting that they express no or very low levels of endogenous TRPC4 and TRPC5 (e.g., Minard et al., Br. J. Pharmacol. 2019).
